# Supplementary material for: No evidence for population-level benefits of polyandry in sharks and rays
Source: PLoS One. 2024 Sep 4;19(9):e0308141. doi: 10.1371/journal.pone.0308141 (PMC11373851; doi:10.1371/journal.pone.0308141)
Supplement: Attachment — Submitted filename: Response to reviewers_2.docx [file pone.0308141.s003.docx]

**Response to reviewers: No evidence for population-level benefits of polyandry in sharks and rays**

We thank the editor and reviewer for their time spent on this manuscript. We are very grateful for both of your comments, and believe that they have greatly improved the quality of the paper. Please find detailed responses to comments below. We hope that you find the changes made sufficient to consider acceptance of the manuscript, as we have to the best of our abilities incorporated all changes suggested by the reviewer into the text.

**Reviewer 1**

**Comment:** Lines 27-28. Please specify if this idea is for the elasmobranch clade or animal kingdom.

**Response:** The sentence has been clarified as requested.

**Comment:** Lines 37-40. I feel that the authors handle this idea a lot, but the topic is not delved into in the discussion, they leave everything to the reader's imagination. In this sense, I have a comment on the discussion.

**Response:** Please see our response to the comment about the discussion.

**Comment:** Lines 43-44. I insist that this idea is no longer under discussion currently, even in elasmobranchs. Also, it doesn't delve into it, I suggest eliminating it.

**Response:** The statement has been eliminated as requested.

**Comment:** Lines 239-242. I suggest exploring this idea a little further in the discussion, I can imagine what they are referring to, but I think it would be better to read it. Since the article is titled "No evidence for population-level benefits of polyandry in sharks and rays" I hope to see some examples in this regard to understand much better how the cancellation of both characteristics (benefits and costs) of the mating system does not necessarily impact the risk of extinction of a species.

**Response:** The current version of the manuscript contains quite a lot of discussion of the costs and benefits of different mating systems. See lines 62-80 and lines 243-255. The fact is that all of these costs and benefits are highly context dependent (as we point out at several points in the text), so we cannot make detailed inferences about specific costs and benefits in specific shark or ray species as we lack the necessary contextual information. The current sections on costs and benefits address to the fullest extent possible how each of these factors could apply to sharks and rays, and provide references that delve deeper into the theoretical basis of those costs and benefits, should the reader be interested.
