# Supplementary material for: No evidence for population-level benefits of polyandry in sharks and rays
Source: PLoS One. 2024 Sep 4;19(9):e0308141. doi: 10.1371/journal.pone.0308141 (PMC11373851; doi:10.1371/journal.pone.0308141)
Supplement: Attachment — Submitted filename: Response to reviewers_3.docx [file pone.0308141.s004.docx]

**Response to reviewers: No evidence for population-level benefits of polyandry in sharks and rays**

The reference list has been checked, and is fully complete. No additional references need to be removed or added to the list. We have made some minor formatting adjustments for consistency as can be seen in the tracked changes document.
